# HIV Partner Service Delivery Among Blacks or African Americans — United States, 2016

**DOI:** 10.15585/mmwr.mm6804a2

**Published:** 2019-02-01

**Authors:** Shubha Rao, Wei Song, Mesfin S. Mulatu, Michele Rorie, Kevin O’Connor, Lamont Scales

**Affiliations:** ^1^Division of HIV/AIDS Prevention, National Center for HIV/AIDS, Viral Hepatitis, STD, and TB Prevention, CDC; ^2^Office of Health Equity, Division of HIV/AIDS Prevention, National Center for HIV/AIDS, Viral Hepatitis, STD, and TB Prevention, CDC.

Identifying persons with human immunodeficiency virus (HIV) infection who are unaware of their infection status, linking them to HIV care, and reducing racial/ethnic disparities are important national HIV prevention goals (*1*). Blacks/African Americans (blacks)* are disproportionately affected by HIV infection in the United States. Although blacks represent 13% of the U.S. population (*2*), in 2017, 44% of diagnoses of HIV infection were in blacks, and the rate of new diagnoses in blacks (41.1 per 100,000 persons) was approximately eight times that of non-Hispanic whites (5.1) (*3*). HIV partner services are offered by health officials to persons with diagnosed HIV infection (index patients) and their sex- or needle-sharing partners, who are notified of their potential HIV exposure and offered HIV testing and related services (*4*). CDC analyzed 2016 data from the National HIV Prevention Program Monitoring and Evaluation system submitted by 59 health departments.^†^ Among 49,266 index patients identified as potential candidates for partner services, 21,191 (43%) were black. The percentage of black index patients interviewed for partner services (76%) was higher than that for all index patients combined (73%). Among the 11,088 black partners named by index patients, 78% were notified of their potential HIV exposure. Fewer than half (47%) of those notified were tested for HIV infection. Among those tested, one in six (17%) received a new HIV diagnosis. The prevalence of newly diagnosed HIV infection was particularly high among black partners who were gay, bisexual, and other men who have sex with men (MSM) (37%) and transgender persons (38%). Effective implementation of partner services is important to identify HIV infection, link patients to care or reengage them in care, and provide prevention services to reduce HIV transmission.

In 2016, CDC funded 61 state and local health departments to implement comprehensive HIV prevention programs, including partner services. CDC analyzed HIV partner services client-level data in the National HIV Prevention Program Monitoring and Evaluation system submitted by 59 health departments. Data were stratified by age group, gender, U.S. Census region,^§^ HIV prevalence,^¶^ and priority population (i.e., MSM, transgender persons, persons who inject drugs, heterosexual males, and heterosexual females).** An index patient is eligible for partner services if he or she is living within the jurisdiction at the time of report. Named partners are eligible for partner services if there is enough information to potentially locate and notify them of their exposure to HIV. Partners with newly diagnosed HIV infection are defined as those who test positive for HIV through partner services–initiated HIV testing and have no evidence of a previous HIV diagnosis reported to the health department surveillance system; recorded in a laboratory report, medical record, or other available data source (e.g., partner services database or records of previous treatment for HIV infection); or recorded in a patient self-report. Partners with a previous diagnosis of HIV infection are those who test positive and have evidence of a previous HIV diagnosis. Data on index patients and partners were extracted from two databases that did not link the race/ethnicity of index patients and partners. Thus, black partners included in this analysis could have been named by index patients of any race/ethnicity. Data on behavioral risk factors used to define the priority population were required for HIV-positive persons and optional for HIV-negative persons. The key outcomes for this analysis include the percentage of black index patients who were interviewed for partner services, HIV status, and the HIV positivity rate among black partners named during the partner services interviews.

Overall, 49,266 index patients were identified as potential candidates for partner services in 2016, including 21,191 (43.0%) who were black (Table 1). The percentage of interviews of black index patients by partner services were higher among those aged 13–19 years (80.6%); females (76.4%); persons residing in the Northeast (80.5%) (excluding U.S. dependent areas); persons residing in low HIV prevalence areas (88.6%), and heterosexual women (92.7%). Among priority populations, percentages of interviews among black index patients by partner services exceeded 90% among heterosexual women (92.7%), heterosexual men (91.5%), and MSM (90.3%); the lowest percentages of interviews among black index patients occurred among those who inject drugs (86.5%) and transgender patients (79.6%).

**TABLE 1:** Number and percentage of all index patients and black index patients offered services through human immunodeficiency virus (HIV) partner services, by demographic characteristics and priority populations — United States,* 2016

Characteristic	All index patients		Black index patients	
	No. (% of total)	No. (%) interviewed	No. (% of total index patients)	No. (%) interviewed
**Total**	**49,266 (100.0)**	**36,037 (73.1)**	**21,191 (43.0)**	**16,153 (76.2)**
**Age group (yrs)^†^**				
13–19	1,002 (2.0)	800 (79.8)	654 (65.3)	527 (80.6)
20–29	15,577 (31.6)	12,086 (77.6)	8,167 (52.4)	6,460 (79.1)
30–39	12,941 (26.3)	9,462 (73.1)	5,223 (40.4)	3,962 (75.9)
40–49	8,569 (17.4)	5,956 (69.5)	2,853 (33.3)	2,075 (72.7)
≥50	10,635 (21.6)	7,545 (70.9)	4,163 (39.1)	3,112 (74.8)
**Gender^§^**				
Male	40,148 (81.5)	29,167 (72.6)	15,853 (39.5)	12,007 (75.7)
Female	7,076 (14.4)	5,308 (75.0)	4,352 (61.5)	3,323 (76.4)
**U.S. Census region^¶^**				
Northeast	5,884 (11.9)	4,696 (79.8)	2,760 (46.9)	2,222 (80.5)
Midwest	4,263 (8.7)	2,586 (60.7)	2,026 (47.5)	1,279 (63.1)
South	28,002 (56.8)	22,387 (79.9)	14,516 (51.8)	11,538 (79.5)
West	10,772 (21.9)	6,031 (56.0)	1,882 (17.5)	1,108 (58.9)
U.S. dependent areas	345 (0.7)	337 (97.7)	7 (2.0)	6 (85.7)
HIV prevalence**				
High	32,920 (66.8)	24,486) (74.4)	1,4084 (42.8)	11,207 (79.6)
Medium	14,876 (30.2)	10,466 (70.4)	6,763 (45.5)	4,685 (69.3)
Medium–low	1,128 (2.3)	812) (72.0)	274 (24.3)	199 (72.6)
Low	342 (0.7)	273 (79.8)	70 (20.5)	62 (88.6)
**Priority population^††^**				
MSM	22,780 (46.2)	19,200 (84.3)	8,155 (35.8)	7,362 (90.3)
Transgender persons	507 (1.0)	374 (73.8)	284 (56.0)	226 (79.6)
Persons who inject drugs	768 (1.6)	640 (83.3)	192 (25.0)	166 (86.5)
Heterosexual men	4,125 (8.4)	3,705) (89.8)	2,395 (58.1)	2,192 (91.5)
Heterosexual women	3,914 (7.9)	3,568 (91.2)	2,523 (64.5)	2,340 (92.7)

**Abbreviation:** MSM = gay, bisexual, and other men who have sex with men.

* Includes U.S. dependent areas of Puerto Rico and the U.S. Virgin Islands.

^†^ Because of missing/invalid data, records were excluded in the column “All index patients” for number of index patients (542; 1.1%) and number interviewed (188; 0.5%) and in the column “Black index patients” for number of black index patients (131; 0.6%) and number interviewed (17; 0.1%).

^§^ Records for transgender persons and other missing/invalid genders were excluded in the column “All index patients” for number of index patients (2,042; 4.1%) and number interviewed (1,562; 4.3%) and in the column “Black Index Patients” for number of black index patients (986; 4.7%) and number interviewed patients (823; 5.1%).

^¶^ *Northeas*t: Connecticut, Maine, Massachusetts, New Hampshire, New Jersey, New York, Pennsylvania, Rhode Island, and Vermont; *Midwest:* Illinois, Indiana, Iowa, Kansas, Michigan, Minnesota, Missouri, Nebraska, North Dakota, Ohio, South Dakota, and Wisconsin; *South:* Alabama, Arkansas, Delaware, District of Columbia, Florida, Georgia, Kentucky, Louisiana, Maryland, Mississippi, North Carolina, Oklahoma, South Carolina, Tennessee, Texas, Virginia, and West Virginia; *West:* Alaska, Arizona, California, Colorado, Hawaii, Idaho, Montana, Nevada, New Mexico, Oregon, Utah, Washington, and Wyoming; *U.S. dependent areas:* Puerto Rico and U.S. Virgin Islands. Two states did not submit data.

** Jurisdictions are grouped according to HIV prevalence and based on the number of persons with diagnosed HIV infection in 2010 (high: ≥20,000; medium: 4,000–19,999; medium–low: 1,000–3,999; and low: <1,000).

^††^ Because of missing risk information, records were excluded in the column “All index patients” for number of index patients (17,172; 34.9%) and number interviewed (8,550; 20.7%) and in the column “Black index patients” for number of black index patients (7,642; 36.1%) and number interviewed (3,867; 23.9%). MSM include males who reported male-to-male sexual contact as well as males who reported both male-to-male sexual contact and injection drug use in the past 12 months. Persons who inject drugs include persons who reported injection drug use in the past 12 months. Heterosexual males include males who only reported heterosexual contact with a female in the past 12 months. Heterosexual females include females who only reported heterosexual contact with a male in the past 12 months. Data on behavioral risk factors used to define the priority population were required for HIV–positive persons and optional for HIV-negative persons.

Among 27,779 partners named by index patients in 2016, a total of 11,088 (39.9%) were black (Table 2). Among named partners who were black, 77.7% (8,616) were notified of their potential HIV exposure. Among partners who were notified, 4,080 (47.4%) were tested for HIV infection. The highest percentages of testing occurred among black partners aged 13–19 years (64.9%); females (60.4%); residents of the Northeast (50.4%); residents of low HIV prevalence areas (72.7%); and heterosexual men (68.4%).

**TABLE 2:** Number and percentage of black partners named, notified, and tested, and new and previous diagnoses of human immunodeficiency virus (HIV) infection through HIV partner services programs, by characteristic — United States,* 2016

Characteristic	Named partners, no. (% by group)	Named partners notified, no. (%)	Notified partners tested, no. (%)	Tested partners with newly diagnosed HIV infection, no. (%)	Tested partners with previously diagnosed HIV infection, no. (%)
**Total**	**11,088 (100.0)**	**8,616 (77.7)**	**4,080 (47.4)**	**690 (16.9)**	**361 (8.8)**
**Age groups (yrs)^†^**					
13–19	248 (2.2)	194 (78.2)	126 (64.9)	15 (11.9)	7 (5.6)
20–29	4,136 (37.3)	3,260 (78.8)	1,837 (56.3)	275 (15.0)	183 (10.0)
30–39	2,484 (22.4)	1,909 (76.9)	1,032 (54.1)	173 (16.8)	89 (8.6)
40–49	1,170 (10.6)	914 (78.1)	504 (55.1)	78 (15.5)	36 (7.1)
≥50	2,113 (19.1)	1,792 (84.8)	483 (27.0)	140 (29.0)	44 (9.1)
**Gender^§^**					
Male	8,563 (77.2)	6,555 (76.6)	3,168 (48.3)	540 (17.0)	285 (9.0)
Female	1,736 (15.7)	1,389 (80.0)	839 (60.4)	98 (11.7)	74 (8.8)
**U.S. Census region^¶^**					
Northeast	1,406 (12.7)	704 (50.1)	355 (50.4)	72 (20.3)	8 (2.3)
Midwest	1,130 (10.2)	650 (57.5)	273 (42.0)	64 (23.4)	7 (2.6)
South	7,848 (70.8)	6,872 (87.6)	3,268 (47.6)	539 (16.5)	335 (10.3)
West	700 (6.3)	388 (55.4)	183 (47.2)	14 (7.7)	11 (6.0)
U.S. dependent areas	4 (0.0)	2 (50.0)	1 (50.0)	1 (100.0)	0 (–)
HIV prevalence**					
High	7,407 (66.8)	6,353 (85.8)	2,964 (46.7)	376 (12.7)	270 (9.1)
Medium	3,388 (30.6)	2,078 (61.3)	1,030 (49.6)	292 (28.3)	85 (8.3)
Medium–low	265 (2.4)	163 (61.5)	70 (42.9)	20 (28.6)	3 (4.3)
Low	28 (0.3)	22 (78.6)	16 (72.7)	2 (12.5)	3 (18.8)
**Priority population^††^**					
MSM	1,731 (15.6)	1,392 (80.4)	839 (60.3)	309 (36.8)	170 (20.3)
Transgender persons	58 (0.5)	40 (69.0)	16 (40.0)	6 (37.5)	1 (6.3)
Persons who inject drugs	17 (0.2)	15 (88.2)	8 (53.3)	2 (25.0)	1 (12.5)
Heterosexual men	542 (4.9)	452 (83.4)	309 (68.4)	69 (22.3)	66 (21.4)
Heterosexual women	467 (4.2)	398 (85.2)	270 (67.8)	65 (24.1)	55 (20.4)

**Abbreviation:** MSM = gay, bisexual, and other men who have sex with men.

* Includes U.S. dependent areas of Puerto Rico and the U.S. Virgin Islands.

^†^ Because of missing/invalid data, records were excluded in the columns for named partners (937; 8.5%); notified partners (547; 6.3%); tested partners (98; 2.4%); newly diagnosed HIV–positive partners (9; 1.3%); and previously diagnosed HIV–positive partners (2; 0.2%).

^§^ Because of missing/invalid data, records for transgender persons and other missing/invalid genders were excluded in the columns for named partners (789; 7.1%); notified partners (672; 7.8%); tested partners (73; 1.8%); newly diagnosed HIV–positive partners (52; 7.5%); and previously diagnosed HIV–positive partners (2; 0.6%).

^¶^ *Northeas*t: Connecticut, Maine, Massachusetts, New Hampshire, New Jersey, New York, Pennsylvania, Rhode Island, and Vermont; *Midwest:* Illinois, Indiana, Iowa, Kansas, Michigan, Minnesota, Missouri, Nebraska, North Dakota, Ohio, South Dakota, and Wisconsin; *South:* Alabama, Arkansas, Delaware, District of Columbia, Florida, Georgia, Kentucky, Louisiana, Maryland, Mississippi, North Carolina, Oklahoma, South Carolina, Tennessee, Texas, Virginia, and West Virginia; *West:* Alaska, Arizona, California, Colorado, Hawaii, Idaho, Montana, Nevada, New Mexico, Oregon, Utah, Washington, and Wyoming; *U.S. dependent areas:* Puerto Rico and U.S. Virgin Islands. Two states did not submit data.

** Jurisdictions are grouped according to HIV prevalence and based on the number of persons with diagnosed HIV infection in 2010 (high: ≥20,000; medium: 4,000–19,999; medium–low: 1,000–3,999; and low: <1,000).

^††^ Because of missing risk information, records were excluded in the columns for named partners (8,273; 74.6%); notified partners (6,319; 73.3%); tested partners (2,638; 64.7%); newly diagnosed HIV–positive partners (239; 34.6%); and previously diagnosed HIV–positive partners (68; 18.8%). MSM include males who reported male-to-male sexual contact as well as males who reported both male-to-male sexual contact and injection drug use in the past 12 months. Persons who inject drugs include persons who reported injection drug use in the past 12 months. Heterosexual males include males who only reported heterosexual contact with a female in the past 12 months. Heterosexual females include females who only reported heterosexual contact with a male in the past 12 months. Data on behavioral risk factors used to define the priority population were required for HIV–positive persons and optional for HIV-negative persons.

Among black partners tested in 2016, 16.9% received a new diagnosis of HIV infection. Newly diagnosed HIV positivity among black partners was higher among persons aged ≥50 years (29.0%); males (17.0%); those residing in the Midwest (23.4%) (excluding U.S. dependent areas); persons residing in medium and medium–low prevalence areas (28.3% and 28.6%, respectively); transgender persons (37.5%); and MSM (36.8%). Among black partners tested, the percentage with previously diagnosed HIV infection was 8.8%. The prevalence of previously diagnosed HIV infection among black partners tested was higher among persons aged 20–29 years (10.0%); males (9.0%); persons residing in the South (10.3%); persons residing in low prevalence areas (18.8%); and heterosexual men (21.4%). Among black MSM partners, 60.3% were tested for HIV.

## Discussion

Among MSM, blacks accounted for 38% of HIV diagnoses in 2017 (*3*). The present analysis found that partner services implemented by CDC-funded health departments interviewed approximately three of four black index patients. Index patients who were black MSM accounted for 45.6% (7,362 of 16,153) of partner services interviews among all black index patients, and approximately 90% of those in this group were interviewed. Fewer than half of all black partners notified of their potential HIV exposure were tested. Among those tested, one in six received a new diagnosis of HIV infection, and one in 11 had a previous diagnosis. The rate of newly diagnosed HIV infection was particularly high among black partners who were MSM (37%) and transgender persons (38%). The high HIV positivity rates among black partners and black MSM partners who were tested are consistent with previous findings that indicate partner services is an effective, high-yield strategy for identifying undiagnosed HIV infections (*5*,*6*). Prevention efforts that promote HIV testing and consistently include partner services might increase early diagnosis and improve HIV-related health outcomes among blacks, particularly among black MSM and transgender persons.

The findings in this report are subject to at least three limitations. First, although CDC provides recommendations outlining the basic elements of partner services (*4*), health department implementation varies considerably. Health departments employ different methods and models for partner services that depend on local legislation and regulations, local service delivery systems, and available resources, including trained disease intervention specialists. Second, the rate of newly diagnosed HIV infection might have been overestimated in those jurisdictions that do not routinely check their laboratory or surveillance records to identify persons with previously diagnosed HIV infection and those jurisdictions with a large proportion of missing data on behavioral risk information. Finally, even though partner services evaluation data requirements are standardized, data collection approaches and systems vary among CDC-funded recipients.

Full and effective implementation of partner services programs to reach all index patients and partners, particularly black MSM and transgender persons, as recommended by the National HIV/Acquired Immunodeficiency Syndrome (AIDS) Strategy, is important to identifying persons who are unaware of their HIV status (*1*). Further, partner services program managers need to ensure that disease intervention specialists have access to all the resources needed to identify and locate partners named by index patients during partner services interviews and to link newly diagnosed partners to HIV medical care. In addition, partner services offer the opportunity to reengage both index patients and previously diagnosed partners who are not in care (*4*). Partner services can also facilitate linkage to HIV preexposure prophylaxis and other prevention services, especially for high risk HIV-negative partners of HIV-positive persons, to reduce their risk of HIV acquisition (*7*). Barriers to effective implementation of partner services and HIV testing include client concerns about compromised confidentiality and fear of negative impacts (e.g., abuse, stigmatization, medical mistrust, and abandonment) (*8*–*10*). Therefore, HIV prevention programs, such as partner services that focus on increasing testing, enhancing linkage to HIV care, reengaging patients with previously diagnosed HIV infection in care, providing prophylactic treatment, and increasing access to support services for blacks, would help to address barriers to service and so reduce onward HIV transmission and HIV-related health disparities.

**Summary**

**What is already known about this topic?**

In 2017, the rate of diagnosis of new human immunodeficiency virus (HIV) infection among blacks/African Americans (blacks) was approximately eight times that of non-Hispanic whites.

**What is added by this report?**

In 2016, 78% of black index patients were interviewed for partner services. However, among black partners, fewer than half were tested for HIV infection, 17% received a new diagnosis of HIV infection, and 9% were previously infected. The prevalence of newly diagnosed HIV infection was particularly high among black partners who were gay, bisexual, and other men who have sex with men (MSM) (37%) and transgender persons (38%).

**What are the implications for public health practice?**

Focusing effective implementation of partner services for blacks, especially for MSM and transgender persons, could lead to reductions in HIV incidence and HIV-related inequities.

## Footnotes

* Persons categorized as blacks/African Americans were not Hispanic or Latino.

† Fifty states, the District of Columbia, Puerto Rico, the U.S. Virgin Islands, and eight metropolitan statistical areas (MSAs) or specified metropolitan divisions (Baltimore, Chicago, Fulton County (Atlanta), Houston, Los Angeles County, New York City, Philadelphia, and San Francisco). In 2016, two health departments did not submit partner services data and were excluded from the analysis.

§ U.S. Census regions (includes MSAs): *Northeast:* Connecticut, Maine, Massachusetts, New Hampshire, New Jersey, New York, New York City (New York), Pennsylvania, Philadelphia (Pennsylvania), and Rhode Island. *Midwest:* Chicago (Illinois), Indiana, Iowa, Kansas, Michigan, Minnesota, Missouri, Nebraska, North Dakota, Ohio, South Dakota, and Wisconsin; *South:* Alabama, Arkansas, Atlanta (Georgia), Baltimore (Maryland), Delaware, Florida, Georgia, Kentucky, Louisiana, Maryland, Mississippi, North Carolina, Oklahoma, South Carolina, Tennessee, Virginia, District of Columbia, and West Virginia. *West:* Alaska, Arizona, California, Colorado, Hawaii, Idaho, Los Angeles (California), Montana, Nevada, New Mexico, Oregon, San Francisco, Utah, Washington, and Wyoming; *U.S. dependent areas:* Puerto Rico and U.S. Virgin Islands.

¶ HIV prevalence is defined based on the number of persons with diagnosed HIV infection in 2010. The jurisdictions are classified based on HIV prevalence as high: ≥20,000; medium: 4,000–19,999; medium–low: 1000–3,999; low: <1,000. https://www.cdc.gov/hiv/pdf/policies/progressreports/cdc-hiv-stateprogressreport.pdf.

** MSM includes males who reported male-to-male sexual contact and those who reported both male-to-male sexual contact and injection drug use in the past 12 months. Persons who inject drugs include persons who reported injection drug use in the past 12 months. Heterosexual males include males who only reported heterosexual contact with a female in the past 12 months. Heterosexual female includes females who only reported heterosexual contact with a male in the past 12 months.
